# Multi-locus Test Conditional on Confirmed Effects Leads to Increased Power in Genome-wide Association Studies

**DOI:** 10.1371/journal.pone.0015006

**Published:** 2010-11-16

**Authors:** Li Ma, Shizhong Han, Jing Yang, Yang Da

**Affiliations:** 1 Department of Animal Science, University of Minnesota, Saint Paul, Minnesota, United States of America; 2 Department of Biological Statistics and Computational Biology, Cornell University, Ithaca, New York, United States of America; 3 Department of Psychiatry, Division of Human Genetics, School of Medicine, Yale University, New Haven, Connecticut, United States of America; Ohio State University Medical Center, United States of America

## Abstract

Complex diseases or phenotypes may involve multiple genetic variants and interactions between genetic, environmental and other factors. Current genome-wide association studies (GWAS) mostly used single-locus analysis and had identified genetic effects with multiple confirmations. Such confirmed single-nucleotide polymorphism (SNP) effects were likely to be true genetic effects and ignoring this information in testing new effects of the same phenotype results in decreased statistical power due to increased residual variance that has a component of the omitted effects. In this study, a multi-locus association test (MLT) was proposed for GWAS analysis conditional on SNPs with confirmed effects to improve statistical power. Analytical formulae for statistical power were derived and were verified by simulation for MLT accounting for confirmed SNPs and for single-locus test (SLT) without accounting for confirmed SNPs. Statistical power of the two methods was compared by case studies with simulated and the Framingham Heart Study (FHS) GWAS data. Results showed that the MLT method had increased statistical power over SLT. In the GWAS case study on four cholesterol phenotypes and serum metabolites, the MLT method improved statistical power by 5% to 38% depending on the number and effect sizes of the conditional SNPs. For the analysis of HDL cholesterol (HDL-C) and total cholesterol (TC) of the FHS data, the MLT method conditional on confirmed SNPs from GWAS catalog and NCBI had considerably more significant results than SLT.

## Introduction

Genome-wide association studies (GWAS) have identified genetic variants associated with a number of complex diseases or phenotypes [1], [2], [3] and some of these variants had confirmations from several studies [1]. Published GWAS studies typically used a single-locus test (SLT), in which each variant is tested individually for association with a specific phenotype. Single-locus analysis may not be the best approach in the presence of confirmed SNP effects, because confirmed effects become a component of random residuals and decrease statistical power for detecting new effects if those true effects are omitted in the analysis. Controlling genetic backgrounds in single-marker tests was well explored in the area of QTL mapping including Zeng's CIM [4] and Jansen's multiple regression method [5]. Roeder et al [6] proposed to use linkage genome scan results in GWAS to achieve higher power. In a recent meta-analysis on smoking behavior, a novel SNP was identified with genome-wide significance related to smoking conditional on a known SNP [7]. Conditional analysis was also used in other meta-analysis in GWAS [8]. In this study, we propose a multi-locus test (MLT) that tests each candidate SNP conditional on confirmed SNPs for GWAS analysis to increase the statistical power for detecting new SNP effects, and we demonstrate the MLT method had increased statistical power relative to SLT using analytical formulae derived in this study and using simulation, case studies, and the Framingham Heart Study (FHS) data.

## Methods

### Predicted Statistical Power of MLT and SLT

The multiple linear regression model for the MLT method can be expressed as:
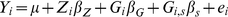
(1)where *μ* = the population mean of the phenotypic values, ***Z*** *_i_* = 1×*p* vector of the *p* covariates for subject *i* (*i* = 1, …, *N*) to account for environment, population stratification, and other factors; ***β*** *_Z_* = *p*×1 vector of the partial regression coefficients of the covariates ***Z*** *_i_*; ***G*** *_i_* = (*G_i_*_,1_, *G_i_*_,2_, …, *G_i,s−1_*) = 1×(*S*−1) vector of the *S*−1 SNP genotypes that were confirmed to be associated with the phenotype for subject *i*, with *G_i,j_* taking values of 0, 1 or 2 according to the number of copies of the minor allele for subject *i* at SNP *j*; ***β*** *_G_* = (*S*−1)×1 vector of partial regression coefficients of *S*−1 conditional SNPs; *G_i,s_* = the genotype value of the candidate SNP; *β_s_* = the partial regression coefficient of the candidate SNP, and *e_i_* = random residual that is assumed to follow N(0, *σ* ^2^) normal distribution. The residual variance of Equation 1 is
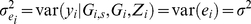
(2)


A standard t-test can be used for testing the significance of the candidate SNP based on testing the following hypotheses, H_0_: *β_s_* = 0, where *β_s_* is the regression coefficient of the candidate SNP and is the *M*th element of ***β*** = (*μ*, ***β*** *_Z_*, ***β*** *_G_*, *β_s_*) and *M* = 1+*p*+*S*. One-sided statistical power was derived in this study for simplicity (two-sided t-test statistical power can be obtained similarly but is not considered here). Using a multiple linear regression framework, the power of the one-sided t-test can be formulated as:
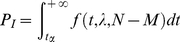
(3)where *t_α_* denotes the α% quantile value for t distribution and *f* denotes the non-central t-distribution with *N*−*M* degrees of freedom and non-centrality parameter:
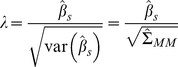
(4)where 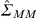 = the element at the *M*th row and *M*th column of variance-covariance matrix 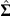, and 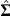 is a *M*×*M* variance-covariance matrix of 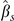 and can be estimated as:
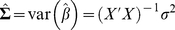
(5)where ***X*** is the design matrix in Equation 1.

For SLT, the statistical model is the same as Equation 1 except that the residual term is now a summation of confirmed effects and random residuals. The residual variance for the SLT model is no longer *σ* ^2^ and has the following mathematical expression:
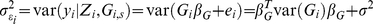
(6)where var(***G*** *_i_*) is calculated based on the ***G*** *_i_* values defined in Equation 1. Equation 6 shows that the residual variance of the SLT model is inflated over the MLT model of Equation 1 due to the fact that confirmed SNP effects are now in the residual term of Equation 6. Using this inflated estimated residual variance, the variance of 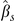 can be estimated by:
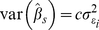
(7)where *c* is the element at the (*p*+2)th row and (*p*+2)th column of matrix 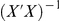 and ***X*** is the design matrix for the regression model of SLT. Therefore, the t-test statistic for SLT does not have a t-distribution but a t-distribution divided by a constant 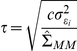.

Similar to Equations 3, the power of the one-sided t-test can be formulated as:
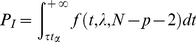
(8)where *t_α_* denotes the α% quantile value for t-distribution with *N*−*p*−*2* degrees of freedom and *f* denotes the non-central t-distribution with *N*−*p*−*2* degrees of freedom and non-central parameter *λ*.

## Results and Discussion

### Evaluation of Statistical Power of MLT and SLT Using Simulation and Real Data

The analytical formulae for statistical power for MLT accounting for confirmed effects and for SLT without accounting for confirmed effects (Equation 3 and Equation 8) were validated by simulated data of 2000 subjects for various effect sizes of the candidate SNP and confirmed SNPs with 10,000 repeats. The phenotypic values were simulated by the summation of a population mean, three additive SNP effects and a random error which followed a standard normal distribution. The three SNPs were simulated under Hardy-Weinberg equilibrium and linkage equilibrium with allele frequencies, 0.3, 0.4 and 0.2. The first two SNPs were assumed to have confirmed effects and the last SNP was assumed to be the candidate SNP. The candidate SNP was tested by the MLT and SLT methods in each simulation. Empirical power was calculated as the proportion of significant results from all 10,000 simulation results. We fixed the effects of the two confirmed SNPs as 0.3 and 0.2 standard deviation of residuals (SD) and varied the effect of the candidate SNP from 0.04 to 0.2 SD. Simulated statistical power were nearly identical to the predicted power for MLT and SLT based on different candidate SNP effect sizes (Table S1) and on different population sizes (Table S2). With this knowledge of the power formulae being correct, predicted statistical power for MLT and SLT were calculated for various effect sizes of the confirmed SNPs (Table 1).

**Table 1:** Power comparison between MLT (Power I) and SLT (Power II) with various conditional SNP effect sizes and constant effect size of 0.1 SD for the candidate SNP.

Effect Size	0.1	0.2	0.3	0.4	0.5	0.6	0.7	0.8	0.9	1.0
Power I	0.713	0.713	0.713	0.713	0.713	0.713	0.713	0.713	0.713	0.713
Power II	0.710	0.701	0.686	0.665	0.638	0.606	0.568	0.525	0.479	0.429
Improvement	0.003	0.012	0.027	0.048	0.075	0.108	0.146	0.188	0.235	0.284

**Figure 1:**
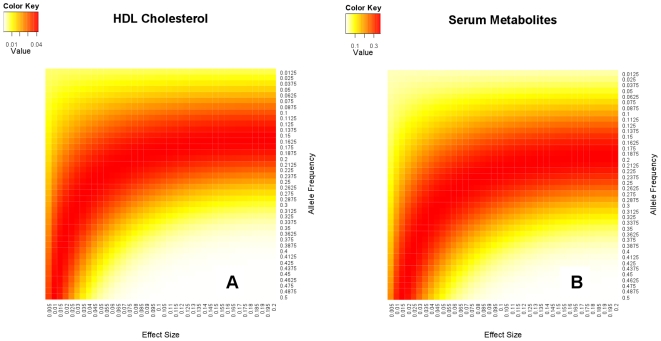
Power improvement of the MLT method conditional on confirmed SNP effects over the SLT method. **a**, HDL Cholesterol. **b**, Serum metabolities.

We further evaluated predicted statistical power using reported effect sizes for some confirmed SNP effects. We collected all reported SNP effects for HDL cholesterol (HDL-C), LDL cholesterol (LDL-C), triglycerides (TG), total cholesterol (TC), and serum metabolites (SM) from the GWAS catalog [1]. The effect sizes and risk allele frequencies of those SNPs were extracted and utilized for the power calculations. After filtering out SNPs in high linkage disequilibrium (LD) by only keeping one SNP with the largest effect size in each high LD region, the final selection of confirmed SNP markers included 22 relatively independent SNPs with effect sizes of 0.07–0.24 SD for HDL-C, 24 SNPs with effect sizes of 0.07–0.35 SD for LDL-C, 13 SNPs with effect sizes of 0.06–0.42 SD for TG, and 19 SNPs with effect sizes of 0.06–0.24 SD for TC. For SM, we extracted five SNPs which explained 5.6 to 36.3 percent of the total phenotypic variation [1], [2]. Conditional on those known significant SNP effects, statistical power of MLT increased over that of SLT by about 4–5% for HDL-C, LDL-C, TG and TC. The pattern of the heatmap for statistical power was similar for these four traits and the heatmap for HDL-C is shown in Figure 1A. Largest improvements were in the region where candidate SNPs had small effect sizes and large allele frequencies or candidate SNPs had medium effect sizes and relatively low allele frequencies (0.1–0.2). The increase in statistical power of MLT over SLT was much larger for SM, varying from 10% to 30%, because of the large effect sizes of the known SNPs (Figure 1B).

For GWAS analysis using real data, true statistical power is not observable but MLT is expected to have more significant results than SLT. To compare observed statistical significance of MLT and SLT, we used the FHS GWAS data (version 2) that had 6575 individuals with SNP genotypes of the 500k SNP panel from dbGAP [9]. Of the 6575 individuals, 6078 individuals had observations on HDL-C and 6431 individuals had observations on TC. From the 500k SNP panel, 432,096 SNP markers with known locations and minor allele frequencies 0.01 or greater were selected and tested. The original cholesterol measures deviated from normality and had outliers. The Box-Cox transformation analysis [10] implemented by the R package [11] showed that the log-transformation was approximately the best transformation to achieve normality for HDL-C and TC. Age, age-squared, cholesterol treatment, blood sugar, body mass index, smoking status, number of cigars smoked, alcohol consumption and sex were adjusted for transformed HDL-C. Blood sugar, body mass index, smoking status, and sex were adjusted for transformed TC. The testing of SNP effects used the generalized least squares version [12] of epiSNP [13]. From the GWAS catalog [1] and NCBI (http://www.ncbi.nlm.nih.gov), we selected six SNP markers (Table 2) with multiple confirmations. These six SNP markers were independent of each other because pairwise correlations measured by R-square among these SNPs were nearly zero. Results showed that MLT had more significant results than SLT for both HDL-C and TC (Table 2). The first two markers in Table 2 had the largest improvement in observed significance (reduced P-value), while the remaining markers only had minor improvement.

**Table 2:** Comparison of P-values of MLT and SLT using the Framingham Heart Study data.

SNP	P-value		Phenotype	Conditional SNPs
	SLT	MLT		
rs1800775	7.06×10^−34^	4.85×10^−35^	HDL-C	rs765547, rs6507945, rs17259942
rs765547	2.19×10^−12^	9.53×10^−13^	HDL-C	rs1800775, rs6507945, rs17259942
rs6507945	9.77×10^−10^	9.51×10^−10^	HDL-C	rs1800775, rs765547, rs17259942
rs17259942	2.77×10^−09^	1.52×10^−09^	HDL-C	rs1800775, rs765547, rs6507945
rs599839	1.23×10^−15^	9.77×10^−16^	TC	rs4245791
rs4245791	3.32×10^−09^	3.21×10^−09^	TC	rs599839

In our analysis, we did not impute genotypic data to fill in missing genotypes so that the MLT test had smaller sample size than SLT. The observed significance should have been larger than observed in Table 2 if the missing genotypes were filled by genotype imputing using software like MACH [14] and BEAGLE [15]. Although improvement in statistical significance could be small in some cases, such improvement could be easily achieved using GWAS analysis software like PLINK [16] and epiSNP [13] that provide options to incorporate covariates. Due to incorporating confirmed effects, MLT has smaller degrees of freedom for residuals and can be less robust than SLT. Fortunately, sample size in typical GWAS studies (thousands) is large enough for incorporating relatively small number of confirmed effects (tens). With more and more confirmed effects reported in the literature, MLT is useful for identifying new genetic variants with smaller effects.

## Supporting Information

Table S1 Power comparison between analytic formulas and simulation for MLT (Power I) and SLT (Power II) with varied candidate SNP effect sizes, constant confirmed effect sizes (0.2 and 0.3 SDs for two confirmed SNPs) and constant sample size (2000). (DOC)

Table S2 Power comparison between analytic formulas and simulation for MLT (Power I) and SLT (Power II) with varied sample sizes, constant candidate SNP effect size (0.1 SD) and confirmed effect sizes (0.2 and 0.3 SDs for two confirmed SNPs). (DOC)
