# Supplementary material for: Multi-locus Test Conditional on Confirmed Effects Leads to Increased Power in Genome-wide Association Studies
Source: PLoS One. 2010 Nov 16;5(11):e15006. doi: 10.1371/journal.pone.0015006 (PMC2982824; doi:10.1371/journal.pone.0015006)
Supplement: Table S1 — Power comparison between analytic formulas and simulation for MLT (Power I) and SLT (Power II) with varied candidate SNP effect sizes, constant confirmed effect sizes (0.2 and 0.3 SDs for two confirmed SNPs) and constant sample size (2000). (DOC) [file pone.0015006.s001.doc]

**Table S1. Power comparison between analytic formulas and simulation for MLT (Power I) and SLT (Power II) with varied candidate SNP effect sizes, constant confirmed effect sizes (0.2 and 0.3 SDs for two confirmed SNPs) and constant sample size (2000).**

| Effect Size | 0.04 | 0.08 | 0.12 | 0.16 | 0.2 |
| --- | --- | --- | --- | --- | --- |
| Power I | 0.171^a^(0.175^b^) | 0.523( 0.519) | 0.857 (0.860) | 0.981(0.981) | 0.999(1.000) |
| Power II | 0.157(0.157) | 0.501(0.491) | 0.844(0.845) | 0.978(0.978) | 0.999(0.999) |

‘a’ represents analytical power and ‘b’ represents simulation power.
