# Supplementary material for: Multi-locus Test Conditional on Confirmed Effects Leads to Increased Power in Genome-wide Association Studies
Source: PLoS One. 2010 Nov 16;5(11):e15006. doi: 10.1371/journal.pone.0015006 (PMC2982824; doi:10.1371/journal.pone.0015006)
Supplement: Table S2 — Power comparison between analytic formulas and simulation for MLT (Power I) and SLT (Power II) with varied sample sizes, constant candidate SNP effect size (0.1 SD) and confirmed effect sizes (0.2 and 0.3 SDs for two confirmed SNPs). (DOC) [file pone.0015006.s002.doc]

**Table S2. Power comparison between analytic formulas and simulation for MLT (Power I) and SLT (Power II) with varied sample sizes, constant candidate SNP effect size (0.1 SD) and confirmed effect sizes (0.2 and 0.3 SDs for two confirmed SNPs).**

| Sample Size | 500 | 1000 | 2000 | 5000 |
| --- | --- | --- | --- | --- |
| Power I | 0.246^a^(0.249^b^) | 0.432( 0.433) | 0.713(0.711) | 0.979(0.977) |
| Power II | 0.228(0.214) | 0.409(0.410) | 0.693(0.683) | 0.976(0.974) |

‘a’ represents analytical power and ‘b’ represents simulation power.
